# Report of an outbreak of enterovirus disease in a neonatal intensive care unit and a systematic review of the literature

**DOI:** 10.3934/microbiol.2025009

**Published:** 2025-02-28

**Authors:** Nathan L'Etoile, Lindsay Brim, Susan Coffin, Ericka Hayes

**Affiliations:** 1 Division of Pediatric Infectious Diseases, Department of Pediatrics, Children's Hospital of Philadelphia, Philadelphia, Pennsylvania, USA; 2 Perelman School of Medicine, University of Pennsylvania, Philadelphia, Pennsylvania, USA; 3 Department of Infection Prevention, Children's Hospital of Philadelphia, Philadelphia, Pennsylvania, USA

**Keywords:** enterovirus, outbreak, neonates, infection prevention, healthcare-associated

## Abstract

Neonatal enterovirus infections have the potential to cause devastating illness and death in this vulnerable age group. Existing evidence suggests that the incidence of enteroviral infections in the post-natal period may be higher than previously thought. Because neonates infected with enterovirus are at risk of severe sequelae, and healthcare-associated outbreaks in neonatal settings can occur, enteroviral infection in hospitalized neonates is a serious concern. Thus, it is essential to conduct surveillance for these infections and to deploy robust infection control measures once the virus has been detected in a neonatal care setting. Here, we report an outbreak of enterovirus in a neonatal intensive care unit (NICU) that was rapidly identified and contained, resulting in relatively few cases but requiring temporary closure of the unit. Additionally, we present our review of the literature describing the characteristics of enteroviral outbreaks in NICU and nursery settings to compare published outcomes of outbreaks to those of our outcome.

## 1. Introduction

Non-polio enteroviruses are known to cause potentially devastating sequelae in neonates and can spread easily via droplets and contaminated fomites, making this a dangerous cause of viral outbreaks in the neonatal intensive care unit (NICU). Neonatal illness caused by enterovirus ranges from enanthems and exanthems typical of hand-foot-and-mouth disease (HFMD) to life-threatening pancarditis, hepatitis, and coagulopathy. Neonatal diseases may be acquired vertically from the mother, at times with devastating consequences, or post-natally [1]. The case fatality rate for severe cases of infection is high, with one review estimating a mortality of up to 30%–38% in infants with myocarditis [2]. Neonatal enterovirus acquisition may be common, but the percentage of asymptomatic cases in infected infants may be high in the late summer and fall, the time of year during which enteroviruses are thought to be more prevalent. One study testing for enterovirus found that the incidence of enterovirus in children born during the late summer and fall was as high as 12.8%, but 79% of the cases were asymptomatic [3].

Infection prevention and control (IPC) strategies are critical to prevent transmission of enteroviruses within healthcare settings. Because effective treatment options for severe enterovirus remain elusive and understudied, prevention measures to limit spread are essential. Rapid recognition and robust outbreak responses are essential. However, other measures to prevent healthcare-associated spread may be important, including surveillance for sporadic cases, awareness of the community's burden of disease, and visitor screening.

While the clinical spectrum of neonatal enterovirus is well described, there is a relative paucity of reports of outbreaks within nursery and neonatal care settings. Understanding effective IPC strategies and mechanisms of transmission can help clinicians and operation leaders at medical institutions stop the spread of enteroviruses.

In this report, we describe an outbreak of enterovirus that occurred within a large, tertiary care NICU and outline the IPC strategies implemented that stopped the ongoing spread of the virus. Additionally, we present a systematic review of the literature describing healthcare-associated outbreaks of enteroviruses, their clinical outcomes, and the infection prevention strategies utilized to halt the spread of this group of viruses and compare prior outbreak experiences to that of our own.

## 2. Materials and methods

### 2.1. Outbreak reporting

This retrospective case series following an outbreak of four cases of HFMD in infants admitted to the NICU and one employee in a large, quaternary care children's hospital is reported in accordance with the Outbreak Reports and Intervention Studies Of Nosocomial Infection (ORION) guidelines [4]. Patients were included if they had a confirmed case of HFMD, defined as a symptomatic patient with a lesion swab positive for enterovirus by polymerase chain reaction (PCR). The enterovirus PCR utilized at our institution is a lab-developed multi-plex real-time PCR using published primers adapted for use in a TaqMan assay [5]. Electronic medical records were reviewed to document symptomatology, physical exam findings, medical history, laboratory evaluation, complications, and additional clinical outcomes. Additionally, staffing assignments and bed location were also obtained by these means. Infection control procedures were documented from meeting notes collected from the outbreak response group.

The hospital is a large, tertiary acute care, academic children's hospital in a large metropolitan area in the United States. The affected wing of the NICU has only private rooms with private bathrooms, in a total of 23 patient rooms. Our infection prevention team consists of nine infection preventionists, four managers, one director, and one medical director. All were involved in the outbreak response group, as were unit personnel, environmental staff, and hospital leadership.

### 2.2. Systematic review of the literature

We conducted a systematic review of healthcare-associated enterovirus outbreaks in neonatal, NICU, and nursery settings in accordance with Preferred Reporting Items for Systematic Reviews and Meta-Analyses (PRISMA) guidelines [6]. After querying PubMed in November 2023 using the keywords (("enterovirus") OR ("echovirus") OR ("coxsackie")) AND ((NICU) OR (neonatal intensive care unit) OR (nursery) OR (intensive care nursery)) AND ((outbreak) OR (cluster)) for papers published since 1970, after the modern enterovirus naming system was adopted, 104 papers were identified. Following an abstract and title review for relevance by one reviewer (NL), 32 studies were included. Reports were included if they described an outbreak of a non-polio enterovirus in either a NICU, a nursery, or a maternity ward in an acute care setting. Reports were excluded if the full text was not available, the report was written in any language other than English, the report only described a single case of enterovirus, or other viruses were included in the evaluation without the ability to extract enterovirus-specific data. Reports were reviewed by one reviewer (NL) for the virus identified, number of cases, staff and visitor involvement, attack rate, clinical outcomes, infection prevention and control measures implemented, season during which the outbreak occurred, and duration of outbreak. Information was only utilized if directly reported in the manuscript.

### 2.3. Definitions and calculations

An outbreak was defined as more than one case of hospital-associated, laboratory-confirmed (PCR) case of enterovirus in a patient who had been admitted to the hospital for >72 hours without an admission diagnosis of known or expected enterovirus disease (neonatal enterovirus, HFMD, etc.) in the NICU, nursery, or maternity ward. A mean, median, and standard deviation were calculated for patient ages and duration of outbreaks using only articles that reported the relevant data. A range of gestational ages of the affected patients at the time of birth was extracted. The mortality rate was calculated as a percentage of deaths of infected patients from reports that reported this outcome. The attack rate was taken from each article's report, and a range of attack rates was calculated.

## 3. Results

### 3.1. Outbreak report

**Figure 1.**
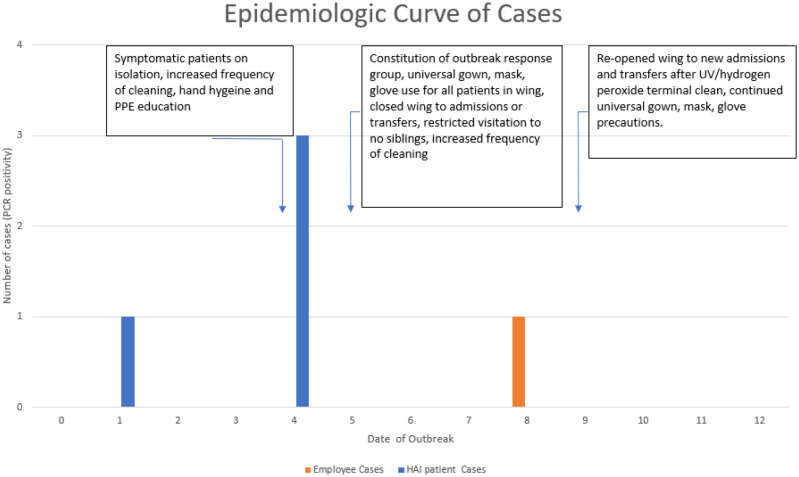
Epidemiologic curve with cases (blue bars) identified during the outbreak. Day 0 corresponds to the date when the index patient developed scalp lesions; subsequently, they tested positive by PCR on Day 1. The employee case was noted on Day 8 of the outbreak. IPC measures are described on the date that they were initiated. “Isolation” refers to the use of gowns, masks, eye protection, and gloves by staff when caring for the affected patients. PPE refers to personal protective equipment.

The index patient was an 8-month-old male born at 25 weeks of gestational age and admitted since birth for management of complications related to prematurity. The patient was first noted to have crusted scalp lesions in early August (Outbreak Day 0). The differential diagnosis for these lesions included folliculitis, pustular melanosis, impetigo, and HFMD. By Day 1, a PCR swab from a lesion was obtained and found to be positive for enterovirus. On Day 3, three other infants were noted to have papulovesicular lesions over the scalp and face in various stages of healing, with some lesions open and others scarred over, consistent with HFMD. PCR swabs from vesicular lesions on all three infants were positive for enterovirus. An epidemic curve annotated with employed IPC measures is provided in Figure 1. Notably, all infants involved were in four different single-bed rooms along the same hallway; all four rooms had dedicated sinks and personal protective equipment donning/doffing areas. Of note, two of the four infants had been under precautions with gowns and gloves for all clinical care for longer than the prior month due to a previous history of colonization with multidrug-resistant organisms. A description of the clinical features of involved infants is provided in Table 1.

### 3.2. Clinical outcomes

The index patient developed a superficial *Staphylococcus aureus* bacterial infection in the scalp lesion that was treated with cefazolin followed by mupirocin. Another patient grew *Klebsiella oxytoca* from a lesion, although this was thought to represent a contaminant without signs of infection and was not treated without sequelae. One patient required escalation in respiratory support six days following the development of symptoms; however, this patient had crusted lesions throughout at the time of escalation, so enterovirus was not suspected to be the cause of the escalation. Three of the four patients developed a fever during this illness; however, no antibiotics were required for any of these infants due to their fever. No other clinical sequelae or work up were noted. Ultimately, the severity of disease was thought to be mild in all affected patients without extensive rash or enanthem. On Day 7 of the outbreak, an employee self-reported symptoms consistent with enterovirus infection and tested positive on Day 9; however, a description of these symptoms was not available for this report.

### 3.3. Exposure investigations

We reviewed medical records to identify shared exposures. We identified a bedside nurse who provided care to the index patient on Days–1 and 0. This individual also cared for two other patients the day before their symptom onset. The affected employee worked until Day 5, wearing appropriate personal protective equipment (PPE) throughout the outbreak. Three of the four infected patients had received care from a single occupational therapist in the days preceding their infection; notably, this therapist cared for the index patient on Day–1 and Day 0.

### 3.4. Infection prevention and control interventions

On Day 3 of the outbreak, IP&C standard precautions were reinforced with staff. All symptomatic patients were placed on contact and droplet precautions (as per the IPC standard at our institution, which includes gowns, gloves, masks, and eye protection). On Day 4 of the outbreak, the affected section of the NICU was placed on universal droplet and contact precautions after recognition of the additional three cases. Concurrently, all admissions and transfers to that area of the NICU were paused for 6 days, corresponding to one incubation period of enterovirus. Sibling visitation was paused, and screening of all adult visitors for viral-like symptoms was reinforced. Environmental services increased cleaning to three times daily for the bed spaces of positive patients, high-touch areas in common areas, and nursing stations. By Day 10, the affected area of the NICU was reopened to new admissions after no further patient positives were identified for 5 days. Following reopening, no further cases of HFMD or enterovirus infection were noted.

**Table 1.** Clinical information regarding patients' infectious course including date of symptom onset, age at positivity, and duration of admission at the time of enterovirus PCR positivity, as well as information regarding transfer from another institution, gestational age at birth, reason for admission, symptoms at onset of enterovirus illness, and sequelae and complications of enterovirus infection. BPD refers to bronchopulmonary dysplasia.

Patient	Date of symptom onset	Age at positivity	Duration of admission	Gestational age at birth	Primary diagnosis/reason for admission	Symptoms at onset	Sequelae and complications
1	8/6/2023	8 months	8 months, transfer to our institution at 1 mo.	25 weeks, 6 days	Pre-maturity, BPD	Rash, fever	Bacterial superinfection of lesions with skin positive for *Staphylococcus aureus*. Infection required systemic and topical antibiotics
2	8/9/2023	5 months	5 months, transfer to our institution at 1 mo.	23 weeks, 6 days	Pre-maturity	Rash, fever 3 days after onset of rash	No antibiotics utilized; however, *Klebsiella oxytoca* grew from a wound.
3	8/9/2023	8 months	7 months	24 weeks, 1 day	Pre-maturity	Rash, irritability	Required escalation in respiratory support 6 days after onset of rash; however, by that time, lesions had crusted over.
4	8/9/2023	8 months	8 months, transfer to our institution at 2 wk.	25 weeks, 4 days	Pre-maturity, BPD	Rash, fever	No antibiotics utilized. Uneventful recovery.

### 3.5. Enterovirus outbreaks within the NICU and hospital nurseries, a review of the literature

**Figure 2.**
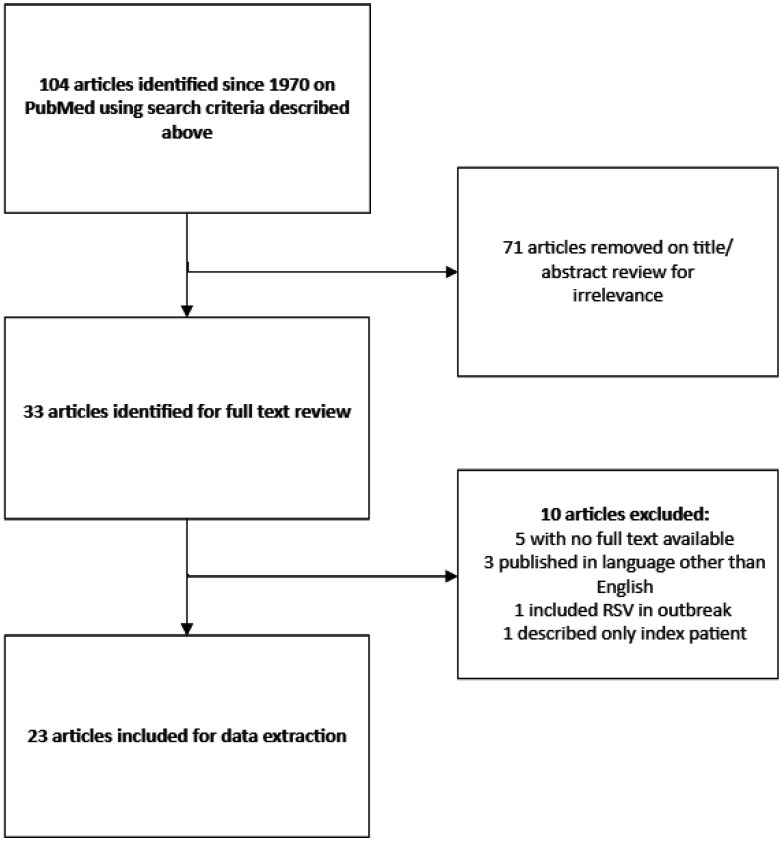
PRISMA diagram denoting search methodologies with excluded articles by title/abstract review and exclusions following full-text review.

Using the described criteria, 32 articles were identified for full-text review (Figure 2). Additional articles were excluded because the full article was not available (n = 5) [7]–[11], the report was in a language other than English (n = 3) [12]–[14], multiple organisms were reported (n = 1) [15], or only an index case was reported/not an outbreak (n = 1) [16]. Thus, 23 articles describing 23 outbreaks were extracted.

Similar numbers of outbreaks were reported from neonatal nurseries (n = 10) and NICUs (n = 11); two outbreaks occurred simultaneously in a NICU and nursery. Most outbreaks (n = 14, 61%) occurred during the summer/fall months only.

257 patients were included, with a median of 10 patients per outbreak (range: 2–23). Nine outbreaks (40%) reported associated employee infections; however, the number of employees impacted was not consistently reported. Among the 126 neonates from whom age at the onset was reported, the median age was 9.5 days. Outbreak duration was available for 16 studies, of which the median duration was 15.5 days (range: 1 day old to 202 days). For seven studies in which the attack rate was either reported or calculable, the attack rate ranged from 9% to 50%. Seven reports involved Echovirus 11, and numerous other enteroviruses were implicated in outbreaks (Table 2). There were 10 deaths reported across all 23 studies out of a total of 257 patients reported (3.9%).

Most studies (n = 17, 74%) described the specific IPC strategies employed to control the outbreaks. Eight studies reported unit closure of infected areas and four reported prophylactic usage of intravenous immune globulin (IVIG). Table 2 outlines the remaining interventions utilized.

**Table 2.** Characteristics of outbreak reports included: author data, number of patients impacted, age of patients, gestational ages of patients, days to resolution, IPC measures implemented, clinical outcomes, month of outbreak, and pathogen identified as cause. Attack rates are included when reported in clinical outcomes (7 studies reported an attack rate). Outbreaks are ordered by publishing date.

Article author and year published	Number of patients impacted	Mean age of patients in days (range)	Gestational age at birth (range)	Days to resolution	Infection control measures implemented	Significant clinical outcomes	Month of outbreak	Pathogen identified
Drew 1978 [17]	10	22 (6–39)	26–40 weeks	30	No measures described	5 infants developed myocarditis. No fatalities reported. Attack rate 50%.	February	Echovirus 11
Faulkner et al. 1973 [18]	7	Not available	Index case: 31 weeks, no further information	Not reported	No measures described	4 infants with abnormal CNS signs. No fatalities reported.	August	Echovirus 17
Eilard et al. 1974 [19]	12	11 (4–47)	29–42 weeks	11	No measures described	Death of index case.	July	Coxsackie B2
Mertens et al. 1982 [20]	6	11 (6–30)	Not reported	14	Strict hand hygiene, isolation	1 infant required escalation of care. No fatalities reported.	October	Echovirus 11
Nagington et al. 1983 [21]	21	5 (3–7)	Not reported	56	Surveillance, prophylactic IVIG	1 death, 5 infants developed meningitis, 6 developed respiratory symptoms, and 1 required kidney resection.	August to October	Echovirus 11
Reiss-Levy et al. 1986 [22]	11	11 (2–56)	29–40 weeks	29	Strict hand hygiene, isolation, environmental decontamination, unit closure	No fatalities reported.	December to January (Southern Hemisphere)	Echovirus 11, Coxsackie B3
Isaacs et al. 1989 [23]	12	28 (16–70)	27–38 weeks	12	Strict hand hygiene	No fatalities reported. Attack rate 29%	October to November	Echovirus 11
Wreghitt et al. 1989 [24]	4	8–9, partial data available	Incomplete data: 31 weeks index case	Not reported	Strict hand hygiene, surveillance, prophylactic IVIG	Death of index case.	June to August	Echovirus 7
Birenbaum et al. 1997 [25]	19	7–202, partial data available	26–40 weeks	Not reported	Strict hand hygiene, isolation, environmental decontamination, cohorting, unit closure	7 infants developed bloody stools, and 4 infants developed abdominal distention. No fatalities reported.	October to November	Echovirus 22
Pasic et al. 1997 [26]	23	Not available	Not reported	15	Prophylactic IVIG, unit closure	7 infants developed severe disease, 3 of which died. Attack rate 34%.	August	Echovirus 4, Echovirus 6
Takami et al. 1998 [27]	4	Not available	Incomplete data, 3/4 full term	17	No measures described	No fatalities reported.	July to August	Echovirus 7
Austin et al. 1999 [28]	2	Not available	Incomplete data, 36 weeks index case	Not reported	Isolation	Index case developed myocarditis. No fatalities reported.	Not reported	Coxsackie B4
Jankovic et al. 1999 [29]	8	7 (5–10)	34–42 weeks	16	No measures described	2 developed severe disease, and 2 deaths were reported.	September	Echovirus 17
Eisenhutt et al. 2000 [30]	4	Not available	Incomplete data, full-term index case	10	Isolation, surveillance, prophylactic IVIG	Death of index case, additional death in asymptomatic patient.	Not reported	Coxsackie A9
Takami et al. 2000 [31]	23	4 (1–17)	Not reported	Not reported	Not reported	No fatalities reported. Attack rate 29%	September 1995 to September 1996	Echovirus 7, Coxsackie B3
Syriopoulou et al. 2002 [32]	20	5.5	Mean 37.8 weeks, two of which were premature	15	Surveillance, Environmental decontamination, unit closure	No fatalities reported. Attack rate 33%.	July	Enterovirus
Apisarnt-hanarak et al. 2005 [33]	13	Not available	Not reported	6	Strict hand hygiene, environmental decontamination, unit closure	No fatalities reported	March	Echovirus 11
Kusuhara et al. 2008 [34]	20	72 (7–180)	Not reported	35	Strict hand hygiene, environmental decontamination, isolation, visitor restrictions, unit closed	No fatalities reported	November to December	Echovirus 18
Huang et al. 2010 [35]	7	42 (22–89)	Not reported	11	Isolation, strict hand hygiene, environmental decontamination, closed unit	4 infants developed encephalitis. No fatalities reported. Attack rate 37%	April to May	Enterovirus 71
Siafakas et al. 2013 [36]	8	18 (6–30)	28–40 weeks	41	Isolation, strict hand hygiene, environmental decontamination, cohorting.	No fatalities reported	July to August	Echovirus 6
Alidjinou et al. 2018 [37]	3	26 (22–29)	30–32 weeks	Not reported	Strict hand hygiene, surveillance	All patients with signs and symptoms of NEC, but no classic features identified. No fatalities reported.	October to November	Echovirus 27
Ho et al. 2020 [38]	10	33 (1–90)	27–40 weeks	23	Isolation, environmental decontamination, visitor precautions.	1 patient with myocarditis, 5 developed meningitis. No fatalities reported.	May to June	Echovirus 11, Coxsackie B3
Xi et al. 2023 [39]	10	27 (7–64)	27–39 weeks	Not reported	Strict hand hygiene, environmental contamination, cohorting, unit closure.	No fatalities reported. Attack rate 33%	July	Echovirus 18

## 4. Discussion

This case series demonstrates an example of an outbreak of enterovirus in a NICU that was contained quickly by a rapid coordinated effort to isolate infants infected with enterovirus and prevent infection of other patients within the unit. Infection prevention methods focused on hand hygiene, universal droplet and contact precautions, environmental decontamination, and unit closure.

The methods used to contain this outbreak have also been consistently used to control other outbreaks described in the literature reviewed. Our outbreak lasted roughly 10 days, shorter than the median duration of 15.5 days reported in this review, which may be related to our IPC measures. Additionally, fewer patients were affected (4) than the median number of patients affected in this literature review (10). Consistent with 40% of the included cases, our outbreak included an employee who was positive via enterovirus PCR. This employee was known to have cared for the index patient, but no other infected infants.

Notably, our outbreak occurred in older patients than those described in the reports reviewed, and their clinical presentation was less severe; also, we did not identify any outbreak that occurred in patients at a similar age range to that of the patients affected in our outbreak. The clinical presentations of our patients were largely limited to HFMD symptoms, all having had a consistent exanthem. While one patient caused concern for a superinfection, this was ultimately treated with topical antibiotics after a brief period of systemic antibiotics. As such, the clinical features of our outbreak were significantly less severe than the constellation of presentations described in the cases reviewed.

The literature review demonstrated a reported mortality of 3.9% associated with infant enterovirus; we included all healthcare-associated cases of enterovirus whereas other reports of mortality have generally focused on severe infection [2]. Importantly, comparisons of the mortality rates between our outbreak and those of reported outbreaks are difficult to make, given the significant age difference between patients affected in our outbreak as compared to those in the reports reviewed. Similarly, the patients included in our outbreak had lower gestational ages (23–27 weeks) than most reported in the systematic review (26–42 weeks). That said, this remains a deadly virus that requires diligent IPC strategies to halt its spread. Interestingly, while 61% of reviewed outbreaks occurred during the summer months, as did our outbreak, the remainder occurred during other seasons. This reinforces the fact that clinicians must be vigilant at all times of the year, with a low threshold to test for enterovirus in a patient or patients with clinically evident disease. Employee involvement in 40% of outbreaks also underpins that employee surveillance and symptom reporting with sick time for affected staff should be observed, as they may serve as vectors during outbreaks.

Four of the reviewed studies reported utilization of IVIG as a preventative measure in asymptomatic, exposed patients [21],[24],[26],[30]. Authors reported that all patients in their respective units were given IVIG, one using IVIG administration as a criterion for discharge [21]. Most studies reported administering a single dose of IVIG; however, one reported a second dose for some infants without citing criteria for a second dose [21]. Two reports described success in preventing infection or reducing symptom severity [21],[26], and one report concluded that IVIG did not prevent subsequent infections, possibly due to the use of a lower IVIG dose (125 mg compared to 250 mg) [24]. One did not report the impact of IVIG on preventing disease or severity [30]. Importantly, none of these reports were designed to evaluate the efficacy of IVIG in preventing or attenuating infection.

While other authors have reported asymptomatic cases involved in outbreaks to demonstrate transmission dynamics, our group did not test those who were asymptomatic, given our retrospective study design. That said, the lack of new cases following the de-escalation of IPC measures contradicts a continued spread of the circulating virus via asymptomatic carriers.

There are limitations in our current report and conclusions drawn from the systematic review. First, the criteria used to search for outbreaks may have been too restricted; however, given the number excluded, this is less likely. Furthermore, the outbreaks incompletely reported surveillance for asymptomatic cases, so the number of infected individuals may be higher than reported. Missing data was present for multiple metrics evaluated, and data was only reported if authors presented clear data in their manuscript. This review demonstrates the opportunity for prospective studies on the impact of IPC measures on preventing and limiting the spread of enterovirus in this vulnerable patient population.

## 5. Conclusions

Enterovirus can cause severe illness in neonates and infants. Our outbreak demonstrated that vigorous IPC measures curtailed the course of a healthcare-associated outbreak. A review of available literature describing outbreaks of various enteroviruses in NICU and nursery settings reinforces that IPC measures are critical in reducing the spread and duration of outcomes, potentially limiting mortality in this vulnerable population.

## Use of AI tools declaration

The authors declare they have not used Artificial Intelligence (AI) tools in the creation of this article.
